# The First Sequenced Carnivore Genome Shows Complex Host-Endogenous Retrovirus Relationships

**DOI:** 10.1371/journal.pone.0019832

**Published:** 2011-05-12

**Authors:** Álvaro Martínez Barrio, Marie Ekerljung, Patric Jern, Farid Benachenhou, Göran O. Sperber, Erik Bongcam-Rudloff, Jonas Blomberg, Göran Andersson

**Affiliations:** 1 Department of Medical Biochemistry and Microbiology, Uppsala University, Uppsala, Sweden; 2 Department of Animal Breeding and Genetics, Biomedical Centre, Swedish University of Agricultural Sciences, Uppsala, Sweden; 3 Section of Virology, Department of Medical Sciences, Uppsala University, Uppsala, Sweden; 4 Department of Neuroscience, Physiology, Uppsala University, Uppsala, Sweden; 5 Department of Genetics and Pathology, Uppsala University, Uppsala, Sweden; Duke University, United States of America

## Abstract

Host-retrovirus interactions influence the genomic landscape and have contributed substantially to mammalian genome evolution. To gain further insights, we analyzed a female boxer (*Canis familiaris*) genome for complexity and integration pattern of canine endogenous retroviruses (CfERV). Intriguingly, the first such in-depth analysis of a carnivore species identified 407 CfERV proviruses that represent only 0.15% of the dog genome. In comparison, the same detection criteria identified about six times more HERV proviruses in the human genome that has been estimated to contain a total of 8% retroviral DNA including solitary LTRs. These observed differences in man and dog are likely due to different mechanisms to purge, restrict and protect their genomes against retroviruses. A novel group of gammaretrovirus-like CfERV with high similarity to HERV-Fc1 was found to have potential for active retrotransposition and possibly lateral transmissions between dog and human as a result of close interactions during at least 10.000 years. The CfERV integration landscape showed a non-uniform intra- and inter-chromosomal distribution. Like in other species, different densities of ERVs were observed. Some chromosomal regions were essentially devoid of CfERVs whereas other regions had large numbers of integrations in agreement with distinct selective pressures at different loci. Most CfERVs were integrated in antisense orientation within 100 kb from annotated protein-coding genes. This integration pattern provides evidence for selection against CfERVs in sense orientation relative to chromosomal genes. In conclusion, this ERV analysis of the first carnivorous species supports the notion that different mammals interact distinctively with endogenous retroviruses and suggests that retroviral lateral transmissions between dog and human may have occurred.

## Introduction

Occasional retrovirus infections in the germ line may lead to inheritance of the proviruses as endogenous retroviruses (ERVs) that are found in all mammals and most vertebrates [1]. Until now, information about the genomic ERV integration landscape and genomic influence has been limited. Here we present the complexity of canine endogenous retroviruses (CfERV) in the dog (Canis familiaris) genome, which is the first sequenced carnivore species. A recent study has connected ERV copy number variation with differentiated replication and infectious potential [2]. Such variation could result from transmission of replication competent retroviruses or even by complementation in *trans* and/or recombination between partially defective ERVs [3], [4]. Retroposons in new genomic contexts are likely to interfere with the host genome function [1]. Retroviral cross-species transmission could likely be pathogenic because the newly infected species has not encountered the retrovirus and thus not developed protection against the infection, *e.g.* HIV pandemic in the human population [5].

However, most ERVs are inactivated by numerous mutations. Host species have co-evolved with ERVs over millions of years and developed multiple defense mechanisms such as co-suppression, CpG methylation and cytidine deamination (reviewed in Jern and Coffin [1]). Active retrotransposition and gammaretrovirus polymorphisms between breeds of domestic pigs and wild boar and also between domestic cats and wild cats have been observed [6], [7]. Evidence for recent endogenization events are also emerging in the primate lineage [8] and in Koala [9].

Retroviruses can be classified into seven genera: *alpha-, beta-, gamma-, delta-, epsilon-, lenti-* and *spuma-like* [1]. However, ERV classification is complicated by interpolation of similarities to infectious retroviruses that have been under selection during replication. Then again, phylogenetic ERV classification using sequence similarity and structural traits has been used successfully [10] towards an updated retrovirus nomenclature [11].

To further our understanding of retrovirus complexity and influence on host genome function and evolution, we analyzed the high-quality dog genome sequence (canFam2, [12]). The dog has emerged as a powerful comparative model for identification of phenotypic traits including genetic disease [13]. Here we analyzed the reference boxer genome (canFam2) using our computer program RetroTector^©^ [14] which is specialized to provirus detection. We show the complex CfERV integration landscape which indicates yet unknown retrovirus restriction or even purging of ERVs from the dog genome and a novel ERV group with potential for retrotransposition and possibly lateral transmission between species.

## Results

### Canine endogenous retroviruses

We screened the dog canFam2 genome for CfERVs using the RetroTector^©^ program [14] and detected 407 proviral “chains”. The average CfERV was 9,187 Kbp and all detected CfERVs (total 3.7 Mbp) comprise about 0.15% of the dog genome (2.5 Gbp). The genomic CfERVs fraction is strikingly lower than in many other species (Table 1).

**Table 1:** Estimated presence of ERVs in different organisms.

Species	Genome Assembly	Chains present	Genome percentage	Assembly depth of coverage*
Zebrafish (*Danio rerio*)	danRer5/4/3	2048	0.8%	6.5–7x
Red jungle fowl (*Gallus gallus*)	gg01	260	0.2%	6.6x
Opposum (*Monodelphis domestica*)	monDom5/4/1	7456	∼2%	7.33x
Dog (*Canis familiaris*)	canFam2	407	<0.15%	7.5x
Mouse (*Mus musculus*)	mm9/8/7	7582	∼2%	7.7x
Rhesus macaque (*Macaca mulatta*)	Mmul_1	2690**	<0.8%	5.1x
Chimpanzee (*Pan troglodytes*)	panTro1/2	2919**	<0.8%	4–6x
Human (*Homo sapiens*)	hg16	3149	0.8%	4–5x

Absolute numbers of detected proviruses in dog (canFam2), chicken (galGal3), zebra fish (danRer4), macaque (rheMac2), chimp (panTro2), human (hg18), opossum (monDom4), and mouse (mm8); adapted from RetroTector^©^ analysis as reported in Blikstad et al. [38].

*Estimated assembly depth from the respective sequencing project.

**RetroTector^©^ may underestimate the real number due to poor quality sequence assembly.

Similarly to the main retrovirus genera detected in human [10], the dog genome contains mainly Gamma-like and Beta-like ERVs (Table S1). The most abundant elements were gammaretrovirus-like (n = 313) with several almost complete chains (13%) and conserved putative proteins (puteins) such as Gag-Pro-Pol (26%) as well as some defective ERVs with conserved Pol (13%). The most common primer binding site (PBS) was complementary to tRNA^Pro^. Four highly mutated CfERVs were related to spumaviruses and only one had detectable flanking long terminal repeats (LTRs), *gag, pol* and *env* genes. The Beta-like elements (n = 28) had more tRNA^Lys^ PBSes. However, a large group of chains (Table S1) could not be classified by RetroTector^©^ and were denoted “unclassified”. Although many different variants were detected, the most common PBS among the “unclassified” proviruses was complementary to tRNA^Pro^.

We detected 44 proviruses with *gag, pro, pol* and *env* genes with putative open reading frames (ORFs). The ORFs are either close to complete or incomplete because they may contain inactivating mutations. Flanking LTRs were detected in 36 of the proviruses. We complemented these findings by calculating the variation in proviral lengths, LTR lengths (5′ and 3′), pairwise LTR divergences, G+C content as well as RetroTector^©^ scores (Fig. S1A–F) for all CfERVs (see below).

### Integration landscape

**Figure 1:**
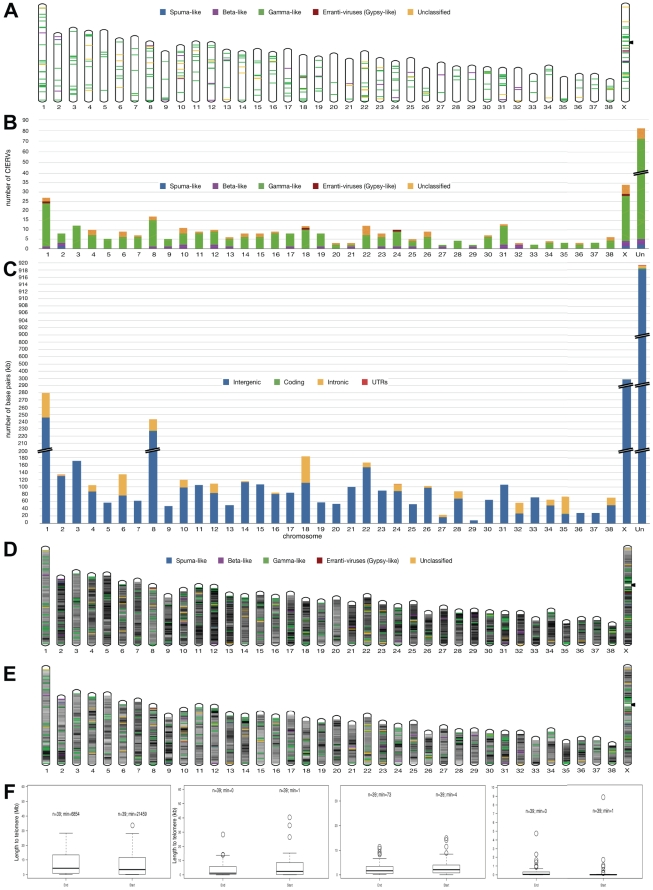
A) Chromosomal distribution of CfERVs. Every CfERV is placed into its chromosomal position rescaled to a megabase (Mb) size bin to be noticeable in the chromosomal picture. A color code is assigned depending on its classified genus. Non-acrocentric chromosomes (chrX) present arrows point at their centromeres. B) Cumulative histogram distinguishing the numbers and genus categories of CfERVs distributed per chromosome. Breaks in bar plots indicate scale changes. C) Cumulative histogram with the CfERV amount of nucleotides contained in exonic, intronic, intergenic or untranslated (UTR) regions per chromosome analyzed. Breaks in bar plots indicate scale changes. D) LINEs (old non-LTR transposable integrations); E) SINE_Cf (newer non-autonomous integrations in dogs). Repeats are grouped in bins to a resolution of 1 Mbp. Relative chromosomal occupancy by these elements of a bin is symbolized by the degree of hue in a grey color scale (darkest, higher). Herein, every CfERV is denoted as specified before. F) Distances to telomeres. The boxplot “Start” describes the distribution of CfERV (or repeat) distances to the telomere at the start of the chromosome, the “End” group is towards the other end of the chromosome. In this graph, the number of elements is represented by ‘n’ (number of chromosomes) and the minimum value in the distribution is ‘min’ for sake of clarity. A number of 0 indicates the fact that an integration of repeat(CfERV) in a telomere exists. Note the different scale in measures (in bp) between images (from left to right): CfERVs contained in each chromosome, LINEs, SINEs-Cf, and repeats without difference of class/type annotated by RepetMasker.

We mapped CfERV integrations on all chromosomes and found a high density of integrations in some regions compared to other nearly empty regions (Fig. 1A). To characterize CfERV integrations into intergenic, intronic, translated and untranslated genomic regions, detected proviruses were mapped to the dog genome and human genes (xref track at UCSC) (Fig. 1C). We confirmed that CfERVs were preferentially located in intergenic and intronic regions with numerous intronic integrations on chromosomes 6 and 18. With a caveat for sequence quality and ERV detection limitations, all chromosomes but 22 (at 6.8 kbp from the q-telomere) appear to lack telomeric CfERVs in contrast to both SINEs and LINEs that appear to be present at telomeres. Apparently, these chains are either not placed in relation to integrations of old (such as LINEs) (Fig. 1D) or young elements (SINEC_Cf) (Fig. 1E) in dogs.

To rule out the possibility that differences in assembly quality account for the higher rate of CfERVs on chromosomes 1 and X, we computed the density of possible ERV-containing sequencing gaps for all chromosomes (Table S2).

We next estimated whether these integrations correlated with chromosome length or with several annotated sets of genes per chromosome, such as protein-encoding genes and non-coding RNA (ncRNA) (Fig. S2A–F). Correlation against chromosome length showed most significance (r^2^ = 0,55; P-value = 3.2×10^−8^). However, weak positive linear correlations were also found against both coding genes and ncRNAs annotated by EnsEMBL and UCSC human-projected genes. The longest chromosomes, 1 and X, did not show linear correlation with any category and were considered outliers (Fig. S2A–F).

### Genomic neighborhood

**Figure 2:**
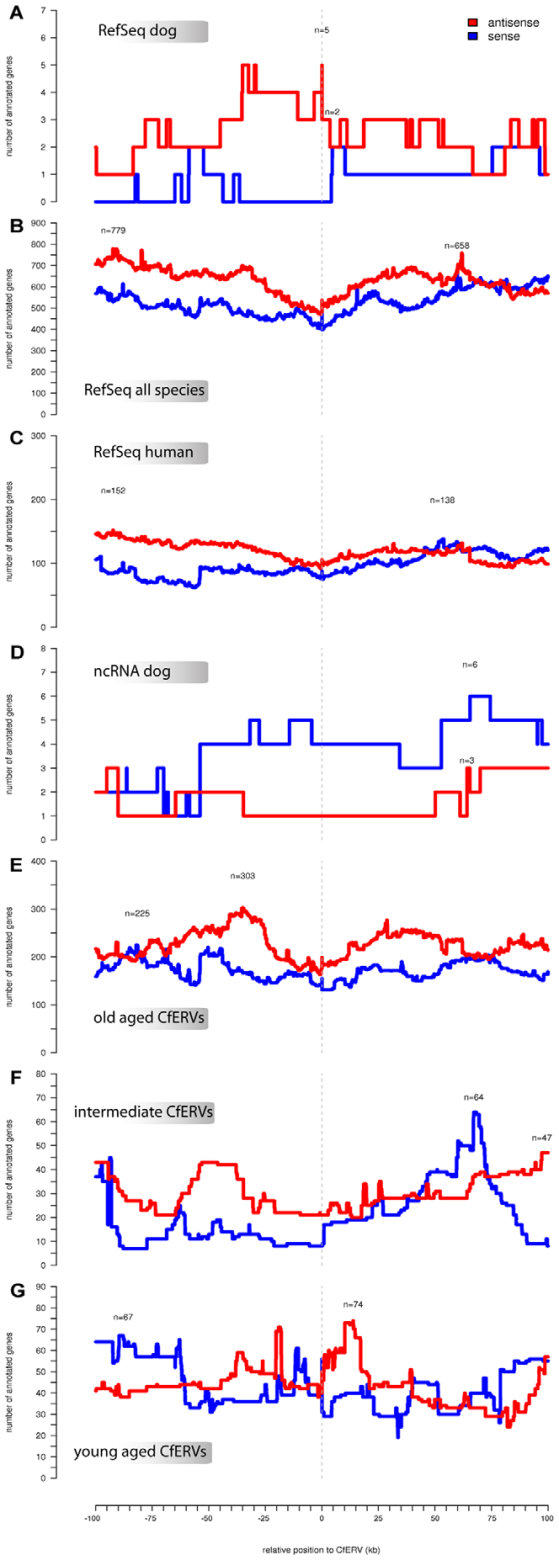
Gene neighboring CfERVs plot. On the x-axis, the distance in bp 3′ and 5′ of the CfERVs in a 200 kb surrounding window [-100 kb, 100 kb] are shown. Position zero refers to the exact location of the CfERV. The presence of genes within each region is shown for the sense (in blue) and antisense (in red) strands. The region with the highest number of genes is marked with the number of genes indicated. A) UCSC RefSeq dog annotated genes; B) including all other UCSC listed species (except dog) annotated genes (xref); C) protein coding genes annotated with only UCSC projections of human genes in the dog genome; D) UCSC xref track annotations counting only ncRNA human genes. E) anciently integrated (>10% LTR divergence); F) intermediately integrated (> = 5% and < = 10%); G) recently integrated (<5%). A visual explanation of the methodology used to calculate the histogram values is sketched in Fig. 3B.

To analyze potential LTR promoter and enhancer functions to adjacent chromosomal genes which have been described for up to 90 kb [15], we collected 100 kb sequences flanking the CfERV integrations. Presence of genes including alternative genomic transcripts, were analyzed in histograms according to their distances to the CfERVs. For this analysis, the longest transcripts were selected and if alternative transcripts overlapped and extended the longest one, these extensions were annexed and this new pseudo-transcript was taken as the final model. The results were divided into “sense” or “antisense” groups depending on CfERV integration relative to chromosomal transcription direction. We then compared the CfERV neighborhood against several datasets: the RefSeq genes annotated at the UCSC genome browser (ref track), for annotated non-canine species in the UCSC browser (xref track), and human genes from the UCSC browser mapped to the dog genome both protein coding and non-coding (Fig. 2A–D).

**Figure 3:**
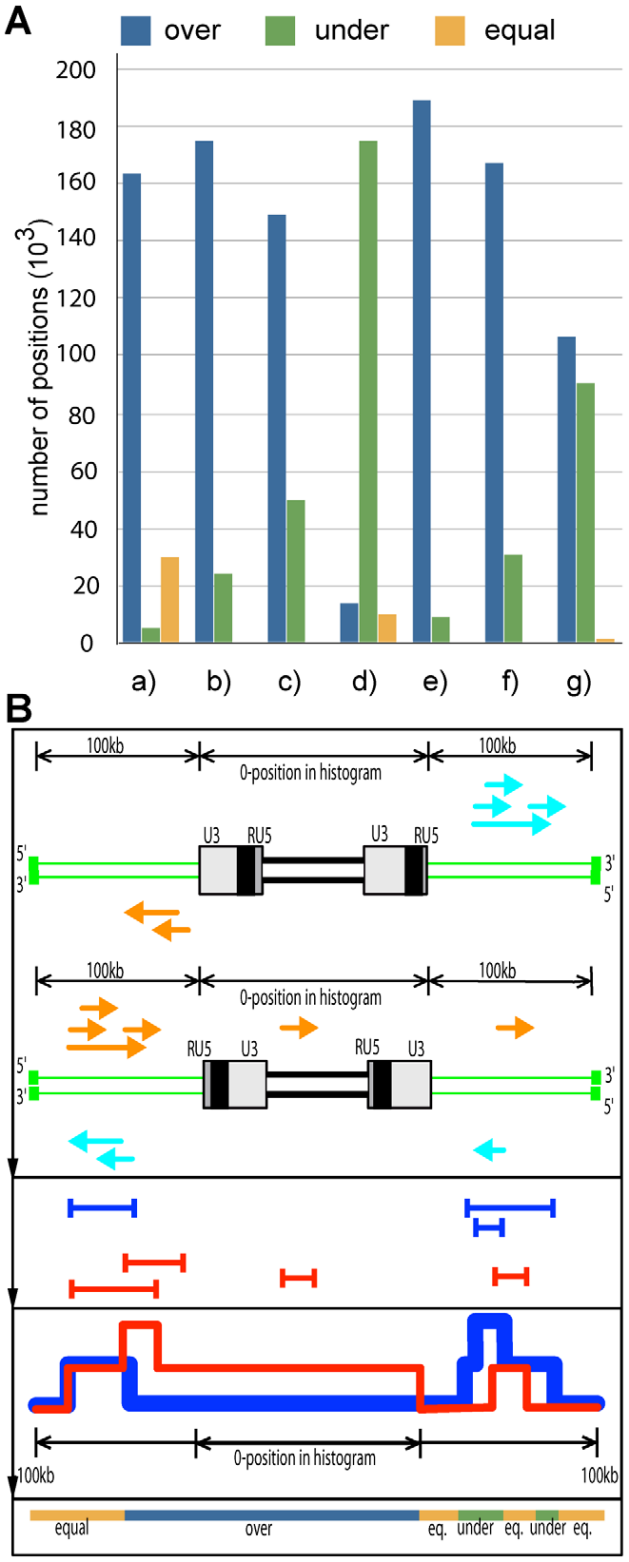
Gene neighborhood statistics. A) Histogram based on the gene vicinity graphs of Fig. 2. Plots indicate the total distribution of genes in the antisense and sense strands. The total number of nucleotides from the longest RefSeq transcript composition overlapping within 200kb context relative to the CfERV integrations and their orientations were measured. Where the presence of a greater number of genes on the antisense –relative to CfERV- strand is found, we classify this in the over-represented category, whereas the presence of more genes on the sense strand –relative to CfERV- is classified as under-represented. This is performed for all regions within 100kb on both sides of every CfERV. From left to right: a) UCSC RefSeq dog annotated genes, b) UCSC listed species (except dog) annotated genes, c) genes annotated with only UCSC projections of human genes in the dog genome, d) UCSC xref track annotations counting only ncRNA human genes, e) only recent integrations (<5% LTR divergence) against UCSC xref track annotations, f) intermediately aged CfERVs (> = 5% and < = 10%), and g) ancient CfERVs (>10%). B) Schematic view explaining the methodology employed to calculate the histogram values of Fig. 2. From top to bottom, from a CfERV integrated in sense (U3-RU5-puteins-U3-RU5), we search a 100kb surrounding for transcripts in the same sense of transcriptional direction (light blue) as well as opposite (dark yellow). Another example, with a CfERV integrated in antisense (RU5-U3-puteins-RU5-U3) is also depicted with transcripts in the opposite (dark yellow) and same relative transcriptional direction (light blue). Each set of overlapping transcripts is composed into a common model of transcripts in antisense (red) and in sense (blue) relative to CfERVs. The blue line has been thickened to highlight the places where both curves take equal values. These models are counted into a −100kb to +100kb histogram where the total of CfERVs detected are centered in position 0. A value for this position suggests that any transcript overlaps any part of the CfERVs, as shown in the example. Finally, for the histogram in Fig. 3A, the x-axis is iterated counting the number of positions where the red curve (antisense) takes a higher value than the blue (sense) depicted in dark blue. These positions sum up to the green bars in the histogram in the A panel, being the opposite situation reflected in the green bars where the blue curve (sense) is higher to the red (antisense). When these two curves take the same exact value, the resultant positions are summed in the dark yellow bars.

Assuming genetic drift and identical LTRs at the time of integration, we separated CfERVs into three groups depending on age estimated from LTR divergence: “young”, “middle” and “old” with less than 5%, 5–10%, and more than 10% LTR divergence, respectively. Using a neutral nucleotide substitution rate of 0.2%/mya [16], a limit of 5% divergence would contain integrations that occurred around 12.5 mya whereas an integration with a 10% divergence would have occurred around 25 mya. Histograms for CfERVs of the three age groups of CfERV loci (Fig. 2E–G) were correlated with genes annotated in the UCSC browser for all other species than dog and with genes which have been projected onto the dog genome, i.e: the xref track. Old CfERVs were found mostly in antisense orientation relative to chromosomal genes when integrated within 70–80 kb from the genes (see Fig. 2E). CfERVs of intermediate age also showed an antisense integration pattern except for a large segment spanning 30 kb (between 42.5 kb and 72.5 kb) (Fig. 2F). Young CfERVs present a more uneven integration pattern with respect to genes in the sense orientation. However, proviruses were more common in antisense 20 kb upstream of the gene, covering the chromosomal promoter region, as well as in sense orientation 6–12 kb downstream of the gene (Fig. 2G).

The number of sense and antisense positions of CfERVs at various distances from the chromosomal gene within 200 kb surrounding context were measured. Positions where the antisense curve takes either a higher (marked as “over” category) or lower value (“under” category) than the corresponding sense value is summarized in Fig. 3A. We also collected the number of CfERVs for which distance values are equal in both the sense and antisense directions. We tested for the independence between the two categories “over” and “under” containing the number of positions where the antisense and sense values are higher than its counterpart. The χ^2^ test yielded a score of 106136.9 with a p-value of 2.2e–16 discarding the independence between the categories and therefore, the normality of their distributions.

Our results revealed CfERVs integrations adjacent to five genes in the antisense direction and two integrations in the sense direction in the promoter region within 5 kb of two other genes (Fig. 2A). These proximal genes were annotated with the corresponding gene ontology terms (Table S3).

The under-annotated UCSC dog RefSeq set currently contains 998 annotated dog genes [12]. A proof for accuracy of this estimation is that the total set of human genes projected onto the dog genome by the UCSC annotation pipeline matched to 19,568 loci. With this gene set projected to the dog genome, independent of CfERV direction relative to chromosomal genes, we discovered 161 genes containing partial CfERVs and 50 CfERVs within 5 kb upstream of annotated genes, in the promoter region, or 5 kb downstream of the 3′UTR.

### Phylogenetic analysis

**Figure 4:**
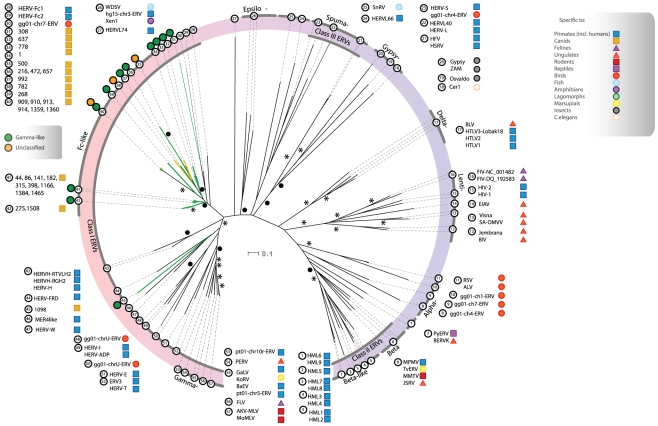
Cluster of the CfERVs with annotated HERV-Fc-like Pol puteins in relation to reference retroviruses. An unrooted NJ tree constructed with a putative evolutionary relationship between HERV-Fc-like CfERV proviruses and their external counterparts. Detected chains are grouped by genus with characteristic colors (green for the gamma-like and yellow for the unclassified). Confidence values to the most deep tree branches are specified over a bootstrapping set of 1000 repetitions. A black asterisk symbolizes a degree of confidence over 90% and solid black circle a higher confidence than 75%.

To perform phylogenetic analyses, we narrowed our CfERV collection to 286 Pol containing integrations of which 219 integrations encoded RT motifs. Finally, only 205 passed our putein quality selection (see materials and methods). When comparing CfERVs with previously described retroviruses in an unrooted tree (Fig. 4), we identified a group that clustered with HERV-Fc-like elements and one outgroup chain (id: 1098) that clustered with the HERV-FRD/MER4like/HERV-W group. After discarding the outgroup element, 24 HERV-Fc-like CfERVs remained and were grouped in separate phylogenies.

### Canine HERV-Fc-like copy-number variation

**Figure 5:**
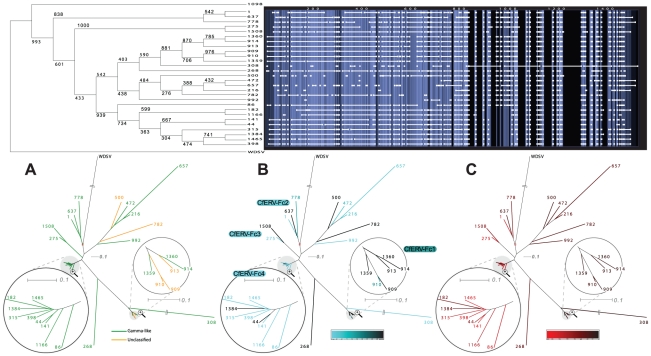
Classification of Fc-like CfERVs with Pol puteins. Upper panel left, rooted tree on the fish WDSV shows the relationship between aligned CfERVs and bootstrapped values (n = 1000). Upper panel right: alignment window where horizontal white bars indicate the presence of aligned viral sequence, larger squared ends represent open gaps, and vertical blue colors indicate the degree of similarity (i.e. light: high, dark: low). Lower panel: three different un-rooted phylograms with WDSV to approximate a root point (red square joint) and zoomed views over dense branches of the tree. A) genus-labeled phylogram, gamma-like and unclassified elements in green and yellow respectively. B) Age classification phylogram, youngest elements in light blue, ancient in dark, undated CfERVs in black. C) Score classification phylogram, highest scoring elements in bright red color. A color scale to measure the variation in tone is provided for both B) and C).

In an attempt to classify CfERVs and to reconstruct the intra-population evolutionary history, we performed a detailed Pol-based phylogenetic analysis with the selected elements (above) and four additional Pol puteins (id: 216, 472, 657 and 992) that had been excluded due to missing RT motifs. In order to identify segmental duplications, we plotted the phylogeny (neighbor joining, 1000 bootstraps) next to the multiple alignment in eBioX [17] (summarized in Fig. 5). To increase the power of our analysis, we compared every candidate CfERV against itself and the other HERV-Fc-like proviruses using dot-matrix analyses without finding significant recombinations.

To identify clusters sharing high similarity and evolutionary history with the HERV-Fc-like proviruses, we analyzed local pairwise identities (Table S4) to similar sequences such as the HERV-Fc1 and -Fc2, as well as a chain from the *Gallus gallus* genome previously detected by RetroTector^©^. In total, four groups were named CfERV-Fc1 to -Fc4 with decreasing age of the youngest group representative estimated from LTR divergences (Fig. 5B). Two proviruses (id: 268 and 308) were not related to any other chain.

The oldest chains are clustered in the CfERV-Fc1 group. Average LTR divergence in the CfERV 910 group suggests that the group originated from an infection around 100-120 mya. The CfERV-Fc4 group represents the most recent expansion (i.e. less than 16.3 mya). The recently integrated CfERV 141 with identical LTRs is the highest scoring and most complete chain with no stop codons and just one frameshift each in *gag* and *env*, and three frameshifts in *pol*. This cluster of nine chains has more than 90% local identity from the branch separation of CfERV 1166 towards the inner tree leaves and 78% on average from the eight innermost elements in the opposite direction towards CfERV 86 (Fig. 5B). CfERVs 141, 1465, and 398 (0, 2 and 2 mya, respectively) are the highest scoring chains in this cluster due to full ORFs except for two stop codons in the CfERV 1465 Gag. Compared to the CfERV-Fc1, this cluster does not seem to have undergone a burst of amplification based on the variation in integration times estimated from LTR divergence. Only CfERVs 44, 182, and 1384 are incomplete.

The CfERV-Fc3 cluster is represented by the fourth highest scoring and intermediate aged (12 mya) CfERV 275. It has *pro* and *env* ORFs, 2 and 5 frameshifts in *gag* and *pol* respectively, and one stop codon in *pol*.

In the CfERV-Fc2 cluster, two chains with very different integration times were present. CfERV 778 (95 mya) has lost the gag gene and accumulated stop codons in both p*ol* and *env* and but not in *pro* which has a frameshift. The LTRs of CfERVs 1 and 778 diverge from the same branch in an LTR phylogeny and network analysis cannot exclude parallel evolution from a common ancestor.

Except for the seemingly oldest and truncated CfERV-Fc1, all CfERV-Fc clusters showed homogeneous genus classification identified by color codes in Fig. 5A. Regarding identity of our selected CfERV-Fc elements to already described Fc-like templates, on average, HERV-Fc1 showed the highest pairwise local identity (55%) whilst HERV-Fc2 and gg01-chr7-ERV showed 44% and 22% identity, respectively.

## Discussion

To further our understanding of ERV integration complexity and effects on host genome function, we have studied the high-quality dog (Canis familiaris) genome sequence of a female boxer (canFam2 [12]). We screened for CfERVs using the platform-independent Java program RetroTector^©^, designed to identify proviral retrovirus sequences in eukaryotic genomes [14]. RetroTector^©^ was efficient in detecting ERVs in human and chimpanzee genomes [18], [19] and automates filtering and categorization of proviral chains using a series of criteria and likelihood tests.

We detected 407 CfERVs corresponding to 0.15% of the entire dog genome. This amount is substantially lower than in human and mouse which have 0,8% and 2% ERVs, respectively (Table 1) using the same selection criteria. The amount of CfERV in dog is as low as the Red Junglefowl (0.2%) and suggests either retrovirus restriction or purging of ERVs by yet unknown mechanisms, The Canids may also have had less retroviral infections compared to primates and rodents. However, the paucity of known extant retroviruses in dogs compared to other mammals as well as the current status of the dog assembly and the limited number of carnivore species sequenced to date preclude firm conclusions regarding mechanisms and processes leading to the low CfERV content observed in dog.

Gamma-like and Beta-like CfERVs were most common in the dog (Table S1). Of the total 407 CfERVs, 44 loci show ORFs with varying conservation. Of these loci, 36 CfERVs had both flanking LTRs, suggesting that around ten percent of the CfERVs are relatively recent integrations. Further, retained functionality of these CfERVs cannot be excluded since complete Gamma-like CfERVs seem to have retained both LTRs to a greater extent whereas related proviruses with only detectable Gag-Pro-Pol have lost their LTRs.

In addition, the Spuma-like and Gypsy-like CfERVs are both represented at low frequencies. However, the former is likely underestimated by RetroTector^©^ due to limited Spuma-related virus references whereas Gypsy-like chains are rare in eutherian genomes [20].

The integration landscape in the dog genome revealed CfERVs mostly in intergenic regions in chromosome X and on all autosomes (Fig 1C). The strongest correlation obtained was between number of CfERV integrations and chromosome length (Fig. S2A). Chromosome 1 is in fact richer in gaps that are likely to be ERV containing compared with the remaining autosomes, suggesting that they may in fact harbor more rather than fewer unassembled ERVs than the rest of the genome (Table S2). The X chromosome is a special case because in the heterogametic sex, meiotic crossing over is constrained entirely to the pseudoautosomal regions (PARs), with the remainder of the chromosome being non-recombining, meaning that the non-PAR X has about half the recombination rate one would expect from obligate crossing over. If efficiency of removal of mobile element insertions increase in regions of higher recombination [21], it would explain the difference in the number of annotations in the sex chromosome and the integration desert in the X_PAR region in dog (chrX:1-6,53 Mb). Furthermore, the analyzed individual is female but the CfERVs integrated in chromosome X would reflect millions of years of canine evolution.

Interestingly, CfERVs integration patterns correlated better with ncRNAs than with protein coding genes (Fig. S2E–F), suggesting a selection against integrations in chromosomal transcription units. This was also supported in our neighborhood analysis of CfERVs with complete LTRs (Fig. 2), where the integration landscape may have retained those a priori selectively neutral integrations. In the neighborhood plots extended with all CfERVs against protein coding genes from different species (Fig. 2A–C), antisense integrations with respect to gene orientation were clearly favored. Surprisingly, when we plotted only the ncRNA genes annotated in the dog genome by the UCSC pipelines (Fig. 2D), the orientation preference changed. This could either imply unknown interactions between ERVs and ncRNAs, or more likely due to under-annotation of ncRNA genes in the dog assembly [12].

CfERV integrations in the close vicinity of genes showed: one integration estimated to about 6 mya, three integrations about 107 mya, and one integration that could not be age estimated. As expected, CfERVs within genes are predominantly in anti-sense orientation relative to the transcriptional direction of the host genes (Fig. 2) assuming that they are less likely to interfere with chromosomal transcription and splicing. These results are in agreement with the integration patterns in both human and mouse [22]. However, conclusions about CfERV interference with gene function as previously hypothesized [23], remains to be drawn.

Among the most interesting CfERVs identified in this study we found a group of 33 HERV-Fc-like proviruses, some recently integrated, that were divided into CfERV-Fc subgroups (Fig. 5). Until now, these elements have exclusively been described as a small group intermediate to ERV-F/H in the primate lineage [24], [25]. Nearly intact HERV-Fc-like proviruses, also previously referred to as possible "midwife elements”, are hypothesized to contribute proteins *in* trans to mobilize similar but less complete ERVs [25]. The CfERV-Fc in dog were surprisingly numerous compared to the characterization of the low copy number HERV-Fc in primates [24]. Interestingly, 10 recently integrated CfERVs had all puteins, albeit some with mutations, as well as both flanking LTRs, making them candidates for spread by complementation *in trans*.

Although highly diverse, young (less or equal to 12 mya), middle (12 to 25 mya) and old chains (more than 25 mya) as dated by LTR divergence, the majority of candidate CfERV-Fc chains (n = 28) could be used in Pol phylogenies. Thus, based on LTR divergence and the most conserved Pol, we have constructed a hypothetical scenario for presence of CfERV-Fc in canids. Our results are consistent with four recent amplification bursts, in the canFam2 dog genome. The four identified groups (i.e: CfERV-Fc1 to -Fc4) presented strong local identities within cluster nodes and 50–60% toward the external branches, which suggested a specific ancestry for each group. Four of the six unclustered CfERVs were devoid of RT motifs complicating their classification. Moreover, local similarities to HERV-Fc templates even after considering different mutation rates along the genome indicated that the CfERV expansion is unlikely to originate from HERV-Fc1.

The phylogenetic analyses suggest that the CfERV-Fc have evolved as exogenous retroviruses that successfully infected the ancestral canid population in bursts, possibly followed by mobilization of endogenous retroviruses. The observed CfERV-Fc sequence differences agree with mutations from extracellular replication and infection by virus strains at different evolutionary stages. It appears that several template sequences have evolved to form the different clusters (Fig. 5) since copy numbers have increased in bursts from extracellular replication rather than retrotransposition. Thus, our proposed CfERV amplification scenario favors a random template master gene model [26] over a strict master gene model [27].

The origin of these proviruses cannot be strictly deduced based on current information. They may either represent new strains of retroviral integrations or alternatively, they may be derived from a founding lateral transfer from other CfERVs or from master ERV-Fc-like sequences. The HERV-Fc2 master may have co-evolved in parallel to the *Canidae* CfERV-Fc and infected primates some 20–32 mya [24]. Thereafter, HERV-Fc1 evolved and infected ancestors of pongids and hominids. According to the low number of infections, ERV-Fc seem to have been unsuccessful in primates but rather more successful in canids, and possibly also in other carnivores. The relatively new HERV-Fc acquisition in baboon [24] is more related to HERV-Fc than to our CfERV-Fc elements and may have infected only cercophitecoids.

In conclusion, the ancestors of *Canidae* has been successful in protecting and/or purging its genome from retroviral integrations but also appears to have been susceptible to certain retrovirus infections. We observed that over time, retrovirus integrations have been selected towards neutral sites. The relative contribution between the permissive selection of these proviruses and the role of domestication of a phenotypically diverse species such as the dog remains unclear. However, our findings support a possibility that some of these proviruses may serve as templates for recombination and that observed proviral ORFs could provide proteins *in trans* to mobilize similar but defective ERVs. The gammaretrovirus-like CfERV-Fc described here, in relatively high copy numbers and with long estimated range of integration time, provides useful insights and understanding of a HERV-Fc-like group that is larger and older than previously considered. Further studies to elucidate functionality and ERV integration polymorphism in multiple dog breeds may define the acquisition pattern of the proviruses and their complex evolutionary relationship with the host in finer detail.

## Materials and Methods

### Screening for canine endogenous retroviruses

The program RetroTector^©^ [14] was used to screen the dog (canFam2) genome [12]. Common retroposons in the dog genome, such as LINE, SINE and MER, were masked prior to the RetroTector^©^ analysis to minimize false positive ERV annotations. Briefly, RetroTector^©^ screens genomic sequence for ERVs by recognizing candidate LTRs followed by detection of internal conserved retroviral consensus motifs while fulfilling distance constraints and then attempts reconstruction of putative ancestral protein sequences, “puteins”, from the three reading frames. The automated process to annotate the puteins and associated ORFs is further described in Sperber *et al.* [14]. The filtering of the proviral chains is based on a series of criteria. The first criterion is to establish a lower threshold score. Randomized data have indicated that scores over 300 separates true from false retroviral chains with a comfortable margin [10]. Other filter criteria are successively applied to allow for only one chain per locus: first by choosing the ERV with a higher presence of different proviral proteins, then, with a higher number of total annotated proteins and finally, according to highest score assigned by RetroTector^©^. All low scoring chains and copies (lower scoring elements in the same locus) are excluded.

### Data collection

All RetroTector^©^ candidate CfERVs were verified by BLAT search [28] (http://genome.ucsc.edu/cgi-bin/hgBlat) against the canFam2 genome. A customized script collected annotations for expression, transcription, translation, genes, retrotransposons, transposons, CpG islands and conservation in 200 Kb sequence contexts surrounding every CfERV locus from the UCSC genome browser datasets [29] (http://genome.ucsc.edu). The RepeatMasker output was downloaded using the UCSC table browser [30], [31] (http://www.repeatmasker.org).

A database of CfERV genes translated into puteins was constructed from the RetroTector^©^ results. A sequence quality control excluded puteins with gaps and sequence errors that caused five or more undetermined amino acids. Similarly, a 15 nt selection criterion was applied to the LTRs. Pol puteins of selected CfERV types were also extracted for intraspecies alignments. With these high quality extant puteins, and protein sequences from other species, we performed interspecies alignments. Phylogenetic analyses include annotated retroviral pol genes from the following clades: canine (Canis familiaris), primate (Homo sapiens, Pan troglodytes, *hylobates*), ungulates i.e. horse (Equus caballus), cattle (Bos taurus), sheep (Ovis aries), murine (Mus musculus), fish (Stizostedion vitreum), and reptile (Python molurus, Crocodylus niloticus). Annotated retroviral reference sequences included in the phylogenies were extracted from GenBank (http://www.ncbi.nlm.nih.gov/) and Jern et al. [10].

Possible CfERV-containing gaps were extracted from the assembly agp files downloaded from GenBank (ftp://ftp.ncbi.nlm.nih.gov/genomes/Canis_familiaris/WGS_assemblies_14July2004/). Clone or contig gaps were excluded because the cause of those is different than a CfERV. Possible CfERV-containing gaps are defined as those which are neither confidently sized too small to contain a complete or partial CfERV element (<1 kbp) nor clearly flanked by fragments of a non-CfERV known repeat element (SINE, LINE, and simple repeats, in particular).

### Data analysis

General statistics of either CfERVs categories (i.e: LTR-divergence), genome wide (i.e: CfERV-neighborhood) or single ERV sequence and the correlations presented in this study were performed using customized analyses tools. The chromosomal visualization was produced using customized scripts and remaining graphs were produced using the R-package.

Multiple alignments were performed using MUSCLE (3.6) [32] with default settings. To outline alignment visualization, we used the eBioX sequence analysis workbench [17]. The NJ phylogenetic trees [33] were constructed using ClustalW (1.83) [34] with pairwise deletions, pairwise distances, Kimura amino acid correction and 1000 bootstraps. Trees were visualized using Dendroscope (v.2.3) [35] and colored using ColorTree [36]. Dot-matrices were calculated with Biopython [37].

## Supporting Information

Figure S1 **Class distribution for the detected CfERVs.** Box-and-whisker plots showing CfERVs divided in genera by (from top to the bottom): A) chain length, LTR length (B) 5′, C) 3′ and D) LTR divergence, E) G+C content and F) the scores assigned by RetroTector^©^ to the grouped proviral chains.
(TIFF)

Figure S2 **Different correlations of CfERVs.** From left to right, top to bottom: against A) chromosomal size; and different gene numbers annotated per chromosome in the B) UCSC dog ref gene database; C) UCSC xref database for human genes mapped (only protein coding); D) EnsEMBL dog core database (with only protein coding); E) UCSC xref database for human genes mapped (only non-coding RNA genes); and F) EnsEMBL dog core database (non-coding RNA genes).
(TIFF)

Table S1 **Class distribution of detected ERVs in the dog genome.** Total number of CfERV distributed in classes, degree of completeness of puteins (if any) for every CfERV detected, number of CfERVs containing either none, any or both of the LTRs, selected structural traits: PBS types (in bold, the most frequent tRNA binding site), NC zinc fingers, immunosuppressive unit(s). Putein and LTR presence cells are empty if no chain belongs to any of the categories shown.
(DOC)

Table S2 **Estimation of CfERV-containing gaps distribution per chromosome in the dog genome.** Chromosomal size and number of reported annotations is displayed alongside the estimation of fragment gaps which possibly contain a total or partial CfERV as well as the variation of Ns annotated in the agp file between the different categories.
(DOC)

Table S3 **Gene list of RefSeq annotated genes in dog overlapped by CfERVs.** Left to right columns:
(DOC)

Table S4 **Identity matrix for the Fc-like chains aligned (** **Fig. 3** **) against Fc-ERVs with templates available (human HERV-Fc1 and HERV-Fc2, and a chicken ERV Fc-like identified as gg01-chr7-ERV).** The alignment is performed with the quality controlled Pol puteins. The upper diagonal lists nucleotide identity and the lower diagonal aminoacid identity.
(TIF)
