# Supplementary figures and images for: The First Sequenced Carnivore Genome Shows Complex Host-Endogenous Retrovirus Relationships
Source: PLoS One. 2011 May 12;6(5):e19832. doi: 10.1371/journal.pone.0019832 (PMC3093408; doi:10.1371/journal.pone.0019832)

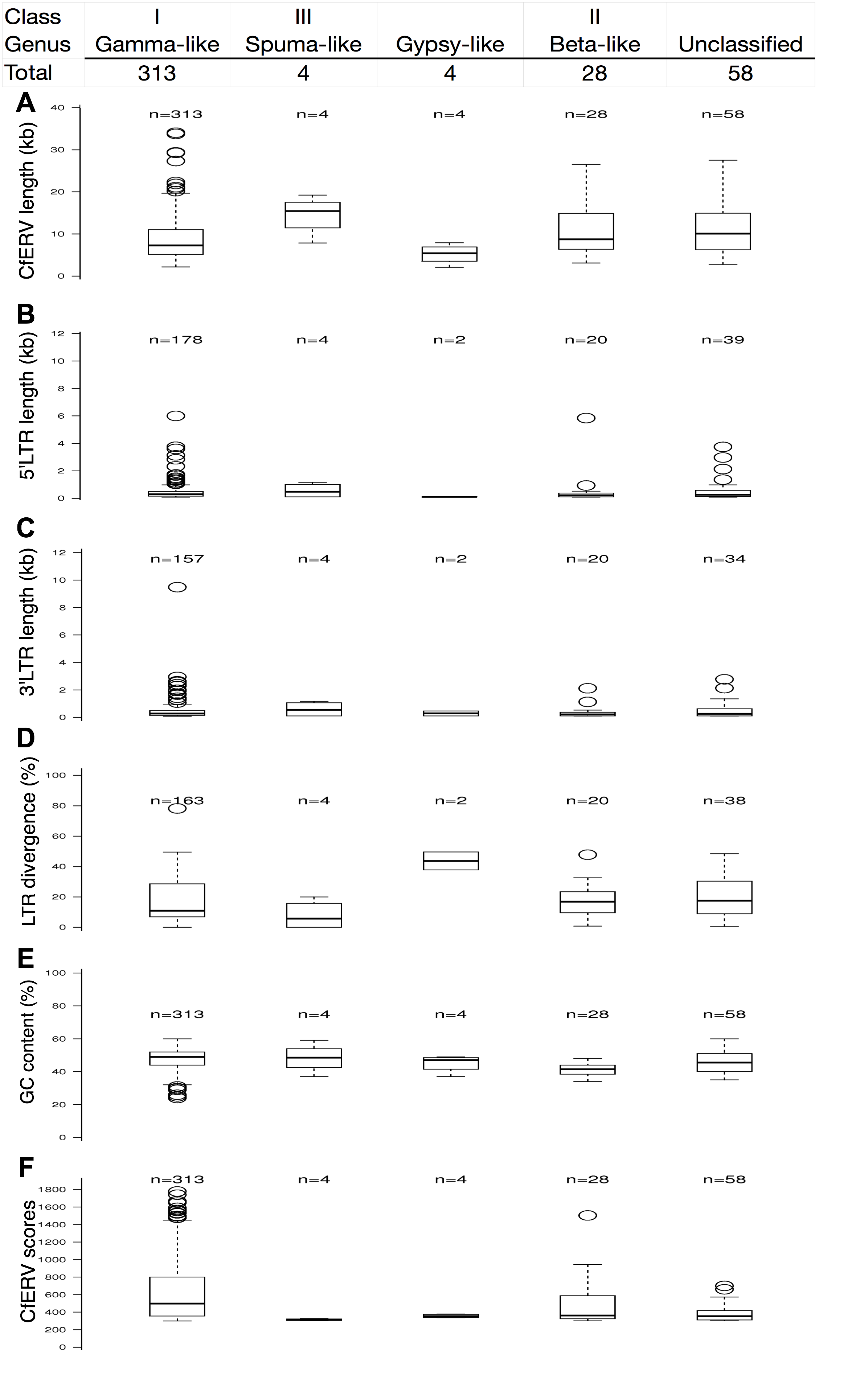

Supplement: Figure S1 — Class distribution for the detected CfERVs. Box-and-whisker plots showing CfERVs divided in genera by (from top to the bottom): A) chain length, LTR length (B) 5′, C) 3′ and D) LTR divergence, E) G+C content and F) the scores assigned by RetroTector© to the grouped proviral chains. (TIFF) [file pone.0019832.s001.tiff]

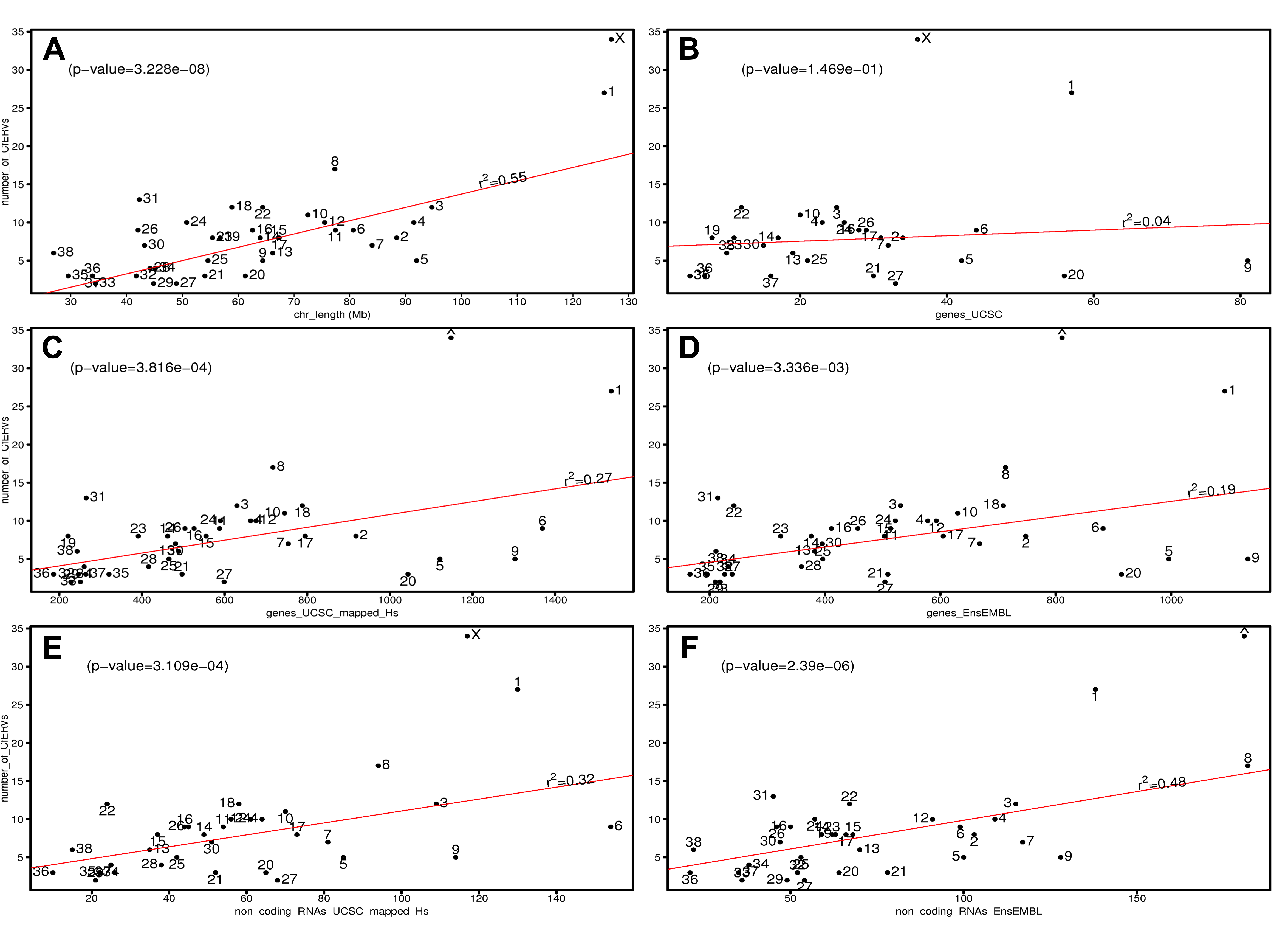

Supplement: Figure S2 — Different correlations of CfERVs. From left to right, top to bottom: against A) chromosomal size; and different gene numbers annotated per chromosome in the B) UCSC dog ref gene database; C) UCSC xref database for human genes mapped (only protein coding); D) EnsEMBL dog core database (with only protein coding); E) UCSC xref database for human genes mapped (only non-coding RNA genes); and F) EnsEMBL dog core database (non-coding RNA genes). (TIFF) [file pone.0019832.s002.tiff]

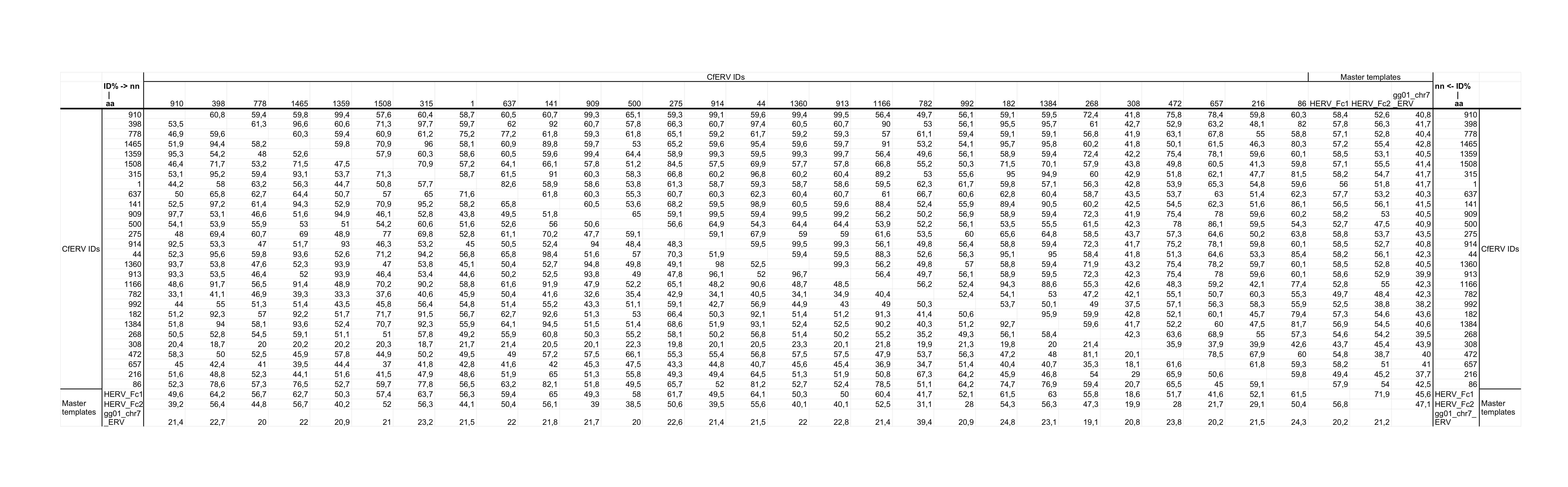

Supplement: Table S4 — Identity matrix for the Fc-like chains aligned ( Fig. 3 ) against Fc-ERVs with templates available (human HERV-Fc1 and HERV-Fc2, and a chicken ERV Fc-like identified as gg01-chr7-ERV). The alignment is performed with the quality controlled Pol puteins. The upper diagonal lists nucleotide identity and the lower diagonal aminoacid identity. (TIF) [file pone.0019832.s006.tif]
