# Supplementary material for: The First Sequenced Carnivore Genome Shows Complex Host-Endogenous Retrovirus Relationships
Source: PLoS One. 2011 May 12;6(5):e19832. doi: 10.1371/journal.pone.0019832 (PMC3093408; doi:10.1371/journal.pone.0019832)
Supplement: Table S1 — Class distribution of detected ERVs in the dog genome. Total number of CfERV distributed in classes, degree of completeness of puteins (if any) for every CfERV detected, number of CfERVs containing either none, any or both of the LTRs, selected structural traits: PBS types (in bold, the most frequent tRNA binding site), NC zinc fingers, immunosuppressive unit(s). Putein and LTR presence cells are empty if no chain belongs to any of the categories shown. (DOC) [file pone.0019832.s003.doc]

Table S1

| Class |  | I | III |  | II |  |
| --- | --- | --- | --- | --- | --- | --- |
| Genus |  | Gamma-like | Spuma-like | Gypsy-like | Beta-like | Unclassified |
| Total |  | 313 | 4 | 4 | 28 | 58 |
|  | Class of puteins |  |  |  |  |  |
| Putein presence (%) | Gag | 2.24% (7/313) |  | 75.00% (3/4) | 3.57% (1/28) | 8.62% (5/58) |
|  | Pro | 0.32% (1/313) |  |  |  |  |
|  | Pol | 12.78% (40/313) |  |  | 7.14% (2/28) | 5.17% (3/58) |
|  | Env | 5.43% (17/313) | 50.00% (2/4) |  | 17.86% (5/28) | 18.97% (11/58) |
|  | Pol-Env | 10.54% (33/313) |  |  | 32.14% (9/28) | 8.62% (5/58) |
|  | Gag-Pol | 5.11% (16/313) |  |  |  | 15.52% (9/58) |
|  | Gag-Env | 0.64% (2/313) |  | 25.00% (1/4) | 3.57% (1/28) | 3.45% (2/58) |
|  | Gag-Pro | 8.31% (26/313) |  |  |  | 8.62% (5/58) |
|  | Pro-Pol | 3.19% (10/313) |  |  | 3.57% (1/28) |  |
|  | Pro-Env | 0.96% (3/313) |  |  |  |  |
|  | Gag-Pol-Env | 3.83% (12/313) |  |  | 3.57% (1/28) | 3.45% (2/58) |
|  | Pro-Pol-Env | 1.60% (5/313) | 25.00% (1/4) |  | 3.57% (1/28) | 1.72% (1/58) |
|  | Gag-Pro-Env | 1.28% (4/313) |  |  |  | 1.72% (1/58) |
|  | Gag-Pro-Pol | 26.20% (82/313) |  |  | 10.71% (3/28) | 10.34% (6/58) |
|  | Gag-Pro-Pol-Env | 13.10% (41/313) |  |  | 7.14% (2/28) | 1.72% (1/58) |
|  | None | 4.47% (14/313) | 25.00% (1/4) |  | 7.14% (2/28) | 12.07% (7/58) |
| LTR presence (None | 5’LTR | 3’LTR | 5’LTR & 3’LTR) | Gag | (0 | 1 | 0 | 6) |  | (2 | 0 | 0 | 1) | (0 | 0 | 0 | 1) | (1 | 1 | 0 | 3) |
|  | Pro | (0 | 0 | 0 | 1) |  |  |  |  |
|  | Pol | (29 | 3 | 0 | 8) |  |  | (2 | 0 | 0 | 0) | (0 | 0 | 0 | 3) |
|  | Env | (0 | 0 | 0 | 17) | (0 | 0 | 0 | 2) |  | (0 | 0 | 0 | 5) | (0 | 0 | 0 | 11) |
|  | Pol-Env | (13 | 1 | 0 | 19) |  |  | (3 | 0 | 0 | 6) | (1 | 0 | 0 | 4) |
|  | Gag-Pol | (11 | 2 | 0 | 3) |  |  |  | (8 | 0 | 0 | 1) |
|  | Gag-Env | (0 | 0 | 0 | 2) |  | (0 | 0 | 0 | 1) | (0 | 0 | 0 | 1) | (0 | 1 | 0 | 1) |
|  | Gag-Pro | (13 | 10 | 0 | 3) |  |  |  | (3 | 2 | 0 | 0) |
|  | Pro-Pol | (7 | 1 | 0 | 2) |  |  | (1 | 0 | 0 | 0) |  |
|  | Pro-Env | (1 | 0 | 0 | 2) |  |  |  |  |
|  | Gag-Pol-Env | (3 | 0 | 0 | 9) |  |  | (0 | 0 | 0 | 1) | (1 | 0 | 0 | 1) |
|  | Pro-Pol-Env | (1 | 0 | 0 | 4) | (0 | 0 | 0 | 1) |  | (0 | 0 | 0 | 1) | (0 | 0 | 0 | 1) |
|  | Gag-Pro-Env | (2 | 1 | 0 | 1) |  |  |  | (0 | 0 | 0 | 1) |
|  | Gag-Pro-Pol | (48 | 19 | 0 | 15) |  |  | (2 | 1 | 0 | 0) | (5 | 1 | 0 | 0) |
|  | Gag-Pro-Pol-Env | (7 | 1 | 0 | 33) |  |  | (0 | 0 | 0 | 2) | (0 | 0 | 0 | 1) |
|  | None | (0 | 0 | 0 | 14) | (0 | 0 | 0 | 1) |  | (0 | 0 | 0 | 2) | (0 | 0 | 0 | 7) |
| PBS |  | 188  (Arg: 9  Asn: 2  Glu: 2  His: 2  Ile: 5  Leu: 5  Met: 6  Phe: 23  **Pro**: 123 Ser: 4  Thr: 4  Trp: 3) | 3  (Arg: 1  Ser: 1  Thr: 1) | 1  **(Phe**: 1) | 17  (Arg: 3  Glu: 1  His: 1  Ile: 2  **Lys**: 9  Met: 1) | 41  (Arg: 5 Asn: 1  Glu: 1  His: 1  Ile: 1  Leu: 3  Lys: 6  Met: 2  Phe: 8  **Pro**: 13) |
| NC zinc finger motifs (1 | 2) |  | 237 (213 | 24) | 0 | 4 (0 | 4) | 10 (6 | 4) | 38 (23 | 15) |
| Immunosuppresive unit (CKS17) |  | 0 | 0 | 0 | 0 | 0 |
| C-term motifs (G-patch | GPY/F) |  | 183 (0 | 183(157)) | 1 (0 | 1(0)) | 0 (0 | 0(0)) | 12 (0 | 12(9)) | 21 (0 | 21(12)) |
