# Supplementary material for: The First Sequenced Carnivore Genome Shows Complex Host-Endogenous Retrovirus Relationships
Source: PLoS One. 2011 May 12;6(5):e19832. doi: 10.1371/journal.pone.0019832 (PMC3093408; doi:10.1371/journal.pone.0019832)
Supplement: Table S2 — Estimation of CfERV-containing gaps distribution per chromosome in the dog genome. Chromosomal size and number of reported annotations is displayed alongside the estimation of fragment gaps which possibly contain a total or partial CfERV as well as the variation of Ns annotated in the agp file between the different categories. (DOC) [file pone.0019832.s004.doc]

Table S2

| **chr** | **chr size (bp)** | **Number of CfERVs annotated** | **Number of gaps** | **Number of fragment gaps** | **Number of fragment gaps CfERV size** | **Number of selected fragment gaps CfERV size (by annotation)** | **Number of Ns (total/fragment/CfERV/selected)** |
| --- | --- | --- | --- | --- | --- | --- | --- |
| 1 | 125616256 | 27 | 1633 | 1631 | 201 | 22 | 4004199/ 999935/656162/ 65273 |
| 2 | 88410189 | 8 | 1183 | 1178 | 156 | 14 | 4070317/ 1063956/816989/ 20750 |
| 3 | 94715083 | 12 | 987 | 983 | 130 | 18 | 3517282/ 512312/309927/ 47907 |
| 4 | 91483860 | 10 | 873 | 871 | 85 | 15 | 3341521/ 338777/163773/ 24194 |
| 5 | 91976430 | 5 | 1225 | 1220 | 132 | 15 | 3514833/ 508388/242604/ 25368 |
| 6 | 80642250 | 9 | 1085 | 1080 | 116 | 14 | 3737911/ 731746/487628/ 27038 |
| 7 | 83999179 | 7 | 873 | 869 | 95 | 7 | 3697510/ 692768/500225/ 129617 |
| 8 | 77315194 | 17 | 858 | 855 | 85 | 9 | 3435536/ 431823/243355/ 12809 |
| 9 | 64418924 | 5 | 1055 | 1050 | 105 | 19 | 3830402/ 824297/599250/ 43199 |
| 10 | 72488556 | 11 | 985 | 983 | 135 | 17 | 3613269/ 610301/374103/ 26182 |
| 11 | 77416458 | 9 | 812 | 810 | 94 | 8 | 3590687/ 588065/418679/ 12450 |
| 12 | 75515492 | 10 | 720 | 719 | 78 | 6 | 3295395/ 293956/149812/ 7324 |
| 13 | 66182471 | 6 | 732 | 729 | 82 | 9 | 3543983/ 540522/378056/ 15804 |
| 14 | 63938239 | 8 | 552 | 551 | 71 | 7 | 3356810/ 355707/251529/ 14563 |
| 15 | 67211953 | 8 | 902 | 901 | 133 | 12 | 3501113/ 499310/313151/ 25693 |
| 16 | 62570175 | 9 | 660 | 657 | 75 | 6 | 3812970/ 809653/679630/ 15342 |
| 17 | 67347617 | 8 | 765 | 763 | 69 | 8 | 3346976/ 344448/184771/ 28212 |
| 18 | 58872314 | 12 | 826 | 824 | 121 | 13 | 3650545/ 647895/452921/ 19519 |
| 19 | 56771304 | 8 | 465 | 463 | 57 | 5 | 3233586/ 231658/137463/ 7791 |
| 20 | 61280721 | 3 | 998 | 996 | 122 | 11 | 3616951/ 613957/377578/ 22319 |
| 21 | 54024781 | 3 | 556 | 555 | 61 | 2 | 3319040/ 317929/203698/ 5427 |
| 22 | 64401119 | 12 | 568 | 566 | 59 | 7 | 3222478/ 220344/114189/ 12877 |
| 23 | 55389570 | 8 | 472 | 471 | 53 | 4 | 3330609/ 329666/227147/ 5317 |
| 24 | 50763139 | 10 | 627 | 626 | 71 | 13 | 3402226/ 400973/251332/ 16970 |
| 25 | 54563659 | 5 | 593 | 589 | 58 | 7 | 3487873/ 483691/351207/ 109588 |
| 26 | 42029645 | 9 | 595 | 593 | 64 | 8 | 3358430/ 356242/232983/ 12811 |
| 27 | 48908698 | 2 | 578 | 577 | 71 | 7 | 3275716/ 274561/147973/ 8686 |
| 28 | 44191819 | 4 | 565 | 562 | 67 | 14 | 3302268/ 299141/180030/ 44959 |
| 29 | 44831629 | 2 | 369 | 368 | 52 | 8 | 3200243/ 199506/134944/ 11868 |
| 30 | 43206070 | 7 | 485 | 484 | 69 | 9 | 3248156/ 247187/145463/ 18284 |
| 31 | 42263495 | 13 | 461 | 459 | 58 | 8 | 3419780/ 417860/319561/ 97213 |
| 32 | 41731424 | 3 | 282 | 281 | 15 | 0 | 3080173/ 79610/24479/0 |
| 33 | 34424479 | 2 | 377 | 375 | 63 | 1 | 3248035/ 246283/171129/ 1079 |
| 34 | 45128234 | 4 | 408 | 406 | 62 | 6 | 3256664/ 254850/173045/ 9196 |
| 35 | 29542582 | 3 | 292 | 291 | 49 | 5 | 3177793/ 177210/114739/ 8427 |
| 36 | 33840356 | 3 | 343 | 342 | 53 | 4 | 3161321/ 160636/ 86578/ 7038 |
| 37 | 33915115 | 3 | 364 | 363 | 52 | 8 | 3189421/ 188694/106020/ 30592 |
| 38 | 26897727 | 6 | 337 | 336 | 53 | 6 | 3231691/ 231018/163846/ 21393 |
| X | 126883977 | 34 | 1683 | 1681 | 216 | 11 | 4639336/ 1634972/1302417/ 159893 |
| ALL | 2445110183 | 325 | 28144 | 28058 | 3388 | 363 | 135263049/18159847/12188386/1172972 |
