# Supplementary material for: The First Sequenced Carnivore Genome Shows Complex Host-Endogenous Retrovirus Relationships
Source: PLoS One. 2011 May 12;6(5):e19832. doi: 10.1371/journal.pone.0019832 (PMC3093408; doi:10.1371/journal.pone.0019832)
Supplement: Table S3 — Gene list of RefSeq annotated genes in dog overlapped by CfERVs. Left to right columns: (DOC) [file pone.0019832.s005.doc]

Table S3

| **CfERV** | **Approx. Integration Time** | **Integration direction** | **Gene** | **Full name** | **Functional role** | **GO annotations** |
| --- | --- | --- | --- | --- | --- | --- |
| 168 | ~6.5 mya | antisense | *CCL28* | Chemokine (C-C motif) ligand 28; also known as mucosae- associated epithelial chemokine (MEC), CCK1 and SCYA2 | Chemotactic activity for resting CD4, CD8 T-cells and eosinophils. This chemokine may play a role in the physiology of extracutaneous epithelial tissues, including diverse mucosal organs. | (PROCESS) [GO:0006935] chemotaxis [GO:0007204] elevation of cytosolic calcium ion concentration [GO:0006955] immune response [GO:0007186] G-protein coupled receptor protein signaling pathway  (FUNCTION) [GO:0005125] cytokine activity [GO:0008009] chemokine activity chemokine activity [GO:0004871] signal transducer activity |
| 441 | ~107 mya | antisense | *HCRTR2* | Hypocretin (orexin) receptor 2; also known as OX2 or OXR2. | G-protein coupled receptor 1 family member involved in the regulation of feeding behavior. | (PROCESS) [GO:0007186] G-protein coupled receptor protein signaling pathway [GO:0007218] neuropeptide signaling pathway [GO:0007631] feeding behavior [GO:0007268] synaptic transmission [GO:0007165] signal transduction  (FUNCTION) [GO:0016499] orexin receptor activity [GO:0008188] neuropeptide receptor activity [GO:0004871] signal transducer activity [GO:0004872] receptor activity [GO:0004930] G-protein coupled receptor activity [GO:0017046] peptide hormone binding |
| 531 | ~108 mya | antisense | *CYP4A37* | cytochrome P450 4A37 | The cytochrome P450 proteins are monooxygenases which catalyze many reactions involved in drug metabolism and synthesis of cholesterol, steroids and other lipids. | (PROCESS) [GO:0055114] oxidation reduction [GO:0006631] fatty acid metabolic process [GO:0001676] long-chain fatty acid metabolic process  (FUNCTION) [GO:0004497] monooxygenase activity [GO:0005506] iron ion binding [GO:0046872] metal ion binding [GO:0018685] alkane 1-monooxygenase activity [GO:0008393] fatty acid (omega-1)-hydroxylase activity [GO:0019825] oxygen binding [GO:0016491] oxidoreductase activity [GO:0009055] electron carrier activity [GO:0020037] heme binding |
| 649 | N.A (5’LTR) | antisense | *CfOLF1* | cOR5I1 olfactory receptor (OR) family 5 subfamily I-like member 1; also known as OR5I1 or OLF1 in human | ORs are G protein-coupled receptors, which characteristically share a 7-transmembrane domain structure with many neurotransmitter and hormone receptors. | (PROCESS) [GO:0007608] sensory perception of smell [GO:0050896] response to stimulus [GO:0007186] G-protein coupled receptor protein signaling pathway [GO:0007165] signal transduction  (FUNCTION) [GO:0004930] G-protein coupled receptor activity [GO:0004871] signal transducer activity [GO:0004872] receptor activity [GO:0004984] olfactory receptor activity |
| 1470 | ~123 mya | antisense | *eda* | Ectodysplasin A, EDA; also known as ED1, ED1-A1, ED1-A2, EDA1, EDA2, or HED | Ectodysplasin is a novel member of the TNF- related ligand family involved in the early epithelial-mesenchymal interaction that regulates ectodermal appendage formation during morphogenesis of these organs. | (PROCESS) [GO:0030154] cell differentiation [GO:0007275] multicellular organismal development [GO:0007165] signal transduction [GO:0007398] ectoderm development [GO:0051092] positive regulation of NF-kappaB transcription factor activity [GO:0001942] hair follicle development  (FUNCTION) [GO:0005164] tumor necrosis factor receptor binding [GO:0005102] receptor binding [GO:0005515] protein binding |
